# Social inclusion, but not exclusion, delays attentional disengagement from direct gaze

**DOI:** 10.1007/s00426-018-1108-2

**Published:** 2018-10-15

**Authors:** Aleksi H. Syrjämäki, Jari K. Hietanen

**Affiliations:** Human Information Processing Laboratory, Faculty of Social Sciences/Psychology, University of Tampere, 33014 Tampere, Finland

## Abstract

The present study investigated whether another person’s direct gaze holds a perceiver’s visuospatial attention and whether social exclusion or social inclusion would enhance this effect. Participants were socially excluded, socially included, or underwent a non-social control manipulation in a virtual ball-tossing game. The manipulation was followed by an attentional disengagement task, in which we measured manual response times in identification of peripheral stimuli shown to the left or right of centrally presented faces portraying direct or downward gaze. Contrary to our hypotheses, the response times were not, in general, longer for direct gaze trials than downward gaze trials, and exclusion did not increase the delay in direct gaze trials. Instead, we discovered that, in the social inclusion group, the response times were longer for direct gaze trials relative to downward gaze trials. Thus, social inclusion might have activated affiliation-related cognitive processes leading to delayed attentional disengagement from faces cueing affiliation.

## Introduction

Social exclusion threatens the fundamental human need to belong (Williams, 2007), lowers mood (Gerber & Wheeler, 2009) and elicits social pain (MacDonald & Leary, 2005). Excluded individuals have an acute need to regain other people’s acceptance (Smart Richman & Leary, 2009; Williams, 2007), and thus they may exhibit affiliative behavior, such as increased conformity (Williams, Cheung, & Choi, 2000) and nonverbal mimicry (Lakin, Chartrand, & Arkin, 2008). Interestingly, when people are socially excluded, they become more efficient in processing of social information, leading to, for instance, increased acuity in identification of facial expressions (Bernstein, Young, Brown, Sacco, & Claypool, 2008), and enhanced memory for social information (Gardner, Pickett, & Brewer, 2000). These findings suggest that excluded individuals allocate a large amount of attentional resources toward socially salient information.

Attentional deployment consists of several different processes such as shifting, engagement, and disengagement of attention (Posner & Petersen, 1990). When an unattended stimulus attracts attention, an individual may shift attention towards it. Some categories of stimuli, such as faces, attract attention more than others so that when several stimuli compete for attention, it is more likely that attention is shifted to these stimuli (Langton, Law, Burton, & Schweinberger, 2008). Biases in the initial shifts of attention toward stimuli belonging to specific categories have been suggested to help in rapid detection of important stimuli (e.g., Cisler & Koster, 2010). After a shift of attention, attention can be engaged by the stimulus, allowing deeper processing of relevant stimulus features. When a novel stimulus suddenly demands attention, attention has to be disengaged from the attended stimulus. Research has revealed that attentional biases can also occur at the stage of attentional disengagement so that disengagement from specific categories of stimuli is delayed. For instance, disengagement is slower from faces than from non-social control pictures (Bindemann, Burton, Hooge, Jenkins, & Haan, 2005), and individuals suffering from anxiety have difficulties in disengaging attention from threatening stimuli (Bar-Haim, Lamy, Pergamin, Bakermans-Kranenburg, & Van IJzendoorn, 2007).

To cope with exclusion, people often allocate attention toward affiliative cues containing information that is particularly important for individuals whose social status is threatened. For instance, in studies where participants have been presented with two faces with different facial expressions, exclusion has been shown to increase the tendency to shift attention toward a smiling face (DeWall, Maner, & Rouby, 2009, Experiment 4; Tanaka & Ikegami, 2015; Xu et al., 2015). It has also been reported that excluded participants are faster than controls in locating smiling faces, but not other emotional faces, from a crowd of faces (DeWall et al., 2009, Experiment 1; but see Tuscherer et al., 2015). Other studies have presented participants simultaneously with several different emotional faces over a period of time, and found that excluded participants, compared to control groups, fixate more on smiling faces, but not other emotional faces (Buckner, DeWall, Maner, & Schmidt, 2010; DeWall et al., 2009, Experiments 2–3; but see Kraines, Kelberer, & Wells, 2018). These findings suggest that excluded individuals tend to shift their attention toward smiling faces, and engage their attention with these faces, possibly because attending to affiliative cues helps them cope with the adverse experience.

Not only facial expressions, but also eye gaze is an important social cue to signal affiliation or exclusion. Direct gaze (gaze directed at the observer’s eye region) indicates that the observer is in the center of the looker’s attention (Conty, George, & Hietanen, 2016). Seeing another’s direct gaze evokes positive affective responses in the perceiver (e.g., Chen, Helminen, & Hietanen, 2017; Chen, Peltola, Ranta, & Hietanen, 2016; Hietanen et al., 2018), and activates brain mechanisms related to approach motivation (Hietanen, Leppänen, Peltola, Linna-aho, & Ruuhiala, 2008). Gaze aversion, on the other hand, is a common way to indicate social exclusion (Williams, Shore, & Grahe, 1998), and it can indeed evoke feelings of exclusion and relational devaluation in the observer (Leng, Zhu, Ge, Qian, & Zhang, 2018; Wirth, Sacco, Hugenberg, & Williams, 2010).

Exclusion has been found to modulate responses to others’ gaze directions. In a recent study, participants were excluded or included in a virtual ball-tossing game Cyberball (see Williams & Jarvis, 2006), which was played ostensibly with other participants present in the laboratory (Lyyra, Wirth, & Hietanen, 2017). After the manipulation, participants judged whether faces with varying gaze directions were looking at them or not. It was discovered that excluded participants, compared to included participants, were biased to view others as portraying direct gaze, suggesting that they viewed others as signaling affiliation with their gaze. However, another study showed that when this game was played ostensibly online with players located in other laboratories, excluded participants tended to judge others as portraying averted gaze instead (Syrjämäki, Lyyra, & Hietanen, 2018). It was suggested that this was because the online setting offered no opportunity for reaffiliation. Exclusion has also been shown to amplify attentional shifts triggered by other people’s gaze. Wilkowski, Robinson, and Friesen (2009) showed that the gaze-cuing effect (the tendency to shift attention toward others’ gaze directions) was larger among individuals with low self-esteem, compared to high self-esteem (Experiment 1), and among participants who had reflected on social exclusion, as compared to those having reflected on inclusion (Experiment 2).

As well as averted gaze, also direct gaze influences perceivers’ attention. Faces portraying direct gaze attract attention more than faces showing other gaze directions (e.g., Böckler, van der Wel, & Welsh, 2014; Conty, Tijus, Hugueville, Coelho, & George, 2006; Lyyra, Astikainen, & Hietanen, 2018; von Grünau & Anston, 1995). Importantly, it has also been suggested that direct gaze holds the perceiver’s visuospatial attention so that attentional disengagement from the face is delayed. This was proposed based on a result that manual response times in detection of peripheral stimuli were longer when participants were shown, in the fixation, a face portraying direct gaze compared to downward gaze or closed eyes (Senju & Hasegawa, 2005). Similarly, a later study reported that delays in saccades to peripheral stimuli were longer from schematic faces suddenly shifting eyes into direct gaze, compared to faces shifting gaze upward or downward (Ueda, Takahashi, & Watanabe, 2014). In another study measuring saccadic latencies and saccadic peak velocities to peripheral targets after pictures of faces with static direct gaze and closed eyes, there was no effect of gaze direction on saccadic latencies, but compatible with the previous studies suggesting delayed attentional disengagement, the peak velocity of the saccades was lower after faces with direct gaze (Dalmaso, Castelli, & Galfano, 2017). Interestingly, however, a recent study found that manual response times in the identification of peripheral stimuli were shorter, not longer, when participants viewed live faces portraying direct gaze, compared to downward gaze (Hietanen, Myllyneva, Helminen, & Lyyra, 2016). The authors suggested that eye contact with the live person increased physiological arousal, and this led to shortened response times after direct gaze stimuli. Thus, the current evidence suggests that only pictures of faces with direct gaze, but not real faces portraying direct gaze, slow down disengagement of attention from the stimulus.

If pictures portraying faces with direct gaze hold perceivers’ visuospatial attention, this effect might be amplified by social exclusion. As exclusion increases allocation of attention to affiliative cues (e.g., DeWall et al., 2009), and amplifies attentional shifts triggered by averted gaze (Wilkowski et al., 2009), it could be expected that the attention holding effect of direct gaze might be particularly strong among excluded individuals. This should lead to further slowing of response times to peripheral target stimuli in the context of direct gaze, as compared to downward gaze.

In the current study, we manipulated participants’ feelings of social exclusion and social inclusion using Cyberball (Williams & Jarvis, 2006), followed by a similar attentional disengagement task as that used by Senju and Hasegawa (2005). In the widely used Cyberball manipulation, participants engage in a virtual ball-tossing game ostensibly with other individuals. Unbeknownst to the participants, the other characters in the game are actually controlled by the computer and are preprogrammed to either include the participants in the game or exclude them from it. Exclusion from this game, compared to inclusion, consistently evokes affective responses associated with social exclusion, such as lowered satisfaction of basic social needs (Hartgerink, van Beest, Wicherts, & Williams, 2015).

A limitation of most studies using the Cyberball manipulation is that they cannot disentangle the effects of social exclusion from the effects of social inclusion. In a typical experiment, excluded participants are compared to included participants, and any differences between the two groups are inferred to reflect effects of social exclusion. However, without a control group it is impossible to determine whether exclusion, inclusion or both caused the observed differences. In the current study, we included a non-social control group, in which participants played a similar ball-tossing game as in the other groups, but the game contained no social interaction (the manipulation has been previously used in Syrjämäki et al., 2018).

The non-social control group also allowed us to investigate the possibility that social inclusion could also slow down attentional disengagement from direct gaze. It has been shown that social inclusion, but not social exclusion, increases interest in mating (Brown, Young, Sacco, Bernstein, & Claypool, 2009; Sacco, Brown, Young, Bernstein, & Hugenberg, 2011). If inclusion can alter social behavior, then it might influence the allocation of attention to social cues as well. Recent evidence shows that the effect of direct gaze, compared to downward gaze, on self-reported arousal is stronger when participants have been primed with affective sentences related to positive social interactions, or social interactions involving the self, compared to negative interactions, or interactions involving other individuals, respectively (McCrackin & Itier, 2018). This suggests that activation of affiliation-related cognitive processes can cause an observer to experience another’s direct gaze as a particularly potent and salient cue. Furthermore, one study found that participants induced with positive mood made more eye contact than participants induced with negative or neutral mood (Natale, 1977). Based on these findings, it seems possible that a positive social experience such as an inclusive social interaction could also modulate responses to others’ gaze, and thus possibly slow down disengagement of attention from faces with direct gaze. On the other hand, only one study has found inclusion in Cyberball causing effects compared to a condition with no manipulation (increased interest in mating; Brown et al., 2009), whereas several studies have found no differences on various measurements when comparing inclusion to non-social control manipulations (Dvir, Kelly, & Williams, 2018; Riva, Williams, Torstrick, & Montali, 2014; Syrjämäki et al. 2018). Thus, exclusion would be more likely to exert an effect on attentional disengagement from direct gaze than social inclusion.

After the social exclusion, social inclusion, or control manipulation, participants completed a task, in which we examined attentional disengagement from faces. We used realistic, computer-generated face stimuli. These kinds of face stimuli have proved useful substitutions for photographs in studies on attention to faces because they provide precise control over many important properties of the stimuli, such as their gaze direction, head orientation, and facial expression (e.g., Becker, Anderson, Mortensen, Neufeld, & Neel, 2011). Similar to earlier research on attentional disengagement from direct gaze, the faces were rotated laterally (Hietanen et al., 2016; Senju & Hasegawa, 2005). In the attentional disengagement task, participants were first shown a face portraying direct or downward gaze in the middle of the computer screen, and after a brief delay (200 ms or 500 ms), a target stimulus appeared on either the left or the right side of the face. Participants were instructed to identify the target stimulus as quickly as possible using one of two keys on a keyboard. We hypothesized that participants in all groups would be slower to identify the target stimuli when presented with a picture of a face portraying direct gaze as compared to downward gaze. Most importantly, we investigated whether social exclusion, and possibly social inclusion, would enhance this effect. We hypothesized that the difference in response times between direct and downward gaze trials would be larger in the social exclusion group, compared to the non-social control group, which would indicate that excluded participants’ attention is particularly strongly held by direct gaze. As described above, there was some basis to expect that the attention holding effect by direct gaze could be enhanced also in the included individuals relative to the control group.

## Method

### Participants

74 participants (26 males, *M*_age_ = 25.4 years, SD_age_ = 6.8) with self-reported normal or corrected to normal vision, and no psychiatric or neurological disorders, volunteered for the experiment. They were randomly assigned to exclusion, inclusion, and non-social control groups. Our aim was to get 20 participants in each condition in the final sample (as suggested by Simmons, Nelson, & Simonsohn, 2011). We excluded a number of participants because they indicated awareness of the deception in the Cyberball manipulation (see “Data analysis” section for details). These participants were replaced to ensure the final sample only consisted of participants not aware of the deception, with sufficient number of participants in each group. The final, analyzed sample after all data exclusions, consisted of 62 participants (*n*_exclusion_ = 21, *n*_inclusion_ = 21, *n*_control_ = 20, 21 males, *M*_age_ = 24.8 years, SD_age_ = 6.0). All participants signed a form of informed consent and were rewarded with either a movie ticket or partial course credit.

### Apparatus

All stimuli were presented on a 19″ LCD monitor with a resolution of 1280 × 1024 and a refresh rate of 60 Hz. Participants’ head position was fixed at 57 cm from the monitor using chin and forehead rests. The experiment was run using E-Prime^®^ 2.0 software. The Cyberball game was presented on a Firefox Internet browser. Participants gave responses using standard keyboards. The right bracket key and the w key were marked with a horizontal line and a vertical line (order counterbalanced across participants) and were used as the response keys in the attentional disengagement task. To align the response keys horizontally, the keyboard was rotated 90° clockwise. To prevent distractions, participants wore acoustic earmuffs during the experiment.

### Social exclusion, social inclusion, and control manipulations

We used three versions of Cyberball 4.0 (Williams & Jarvis, 2006) for the social exclusion, inclusion, and control manipulations. In the inclusion and exclusion conditions, three characters, one controlled by the participant, were throwing a ball with each other. Participants in the inclusion condition received approximately one-third of all tosses. Participants assigned to the exclusion condition only received the ball once from each character in the beginning of the game, and then never again. The game lasted for 30 throws in total. In the control condition, the other two characters were replaced by pictures of baskets, in which the participants were throwing the ball. After each throw, the ball returned to the participant’s character. Participants in the control condition made 10 throws, i.e., the same number as in the inclusion condition. The game pace was adjusted to ensure the length of the game was similar in all conditions.

### Stimuli

The face stimuli in the attentional disengagement task were faces of four virtual characters (two females), created with 3D animation software DAZ Studio. The whole head, including the neck, was displayed. The characters were rotated 20° on a vertical axis. The eyes in the direct gaze stimuli were individually rotated so that the pupils pointed directly at the camera. The eyes in the downward gaze stimuli were rotated similarly on the vertical axis, but they pointed 24.1° down. The stimuli were 11.7° (± 0.4°) high, and 8.1° (± 0.8°) wide. The target stimuli were horizontal and vertical lines, 1.3° of visual angle.

### Attentional disengagement task

**Fig. 1:**
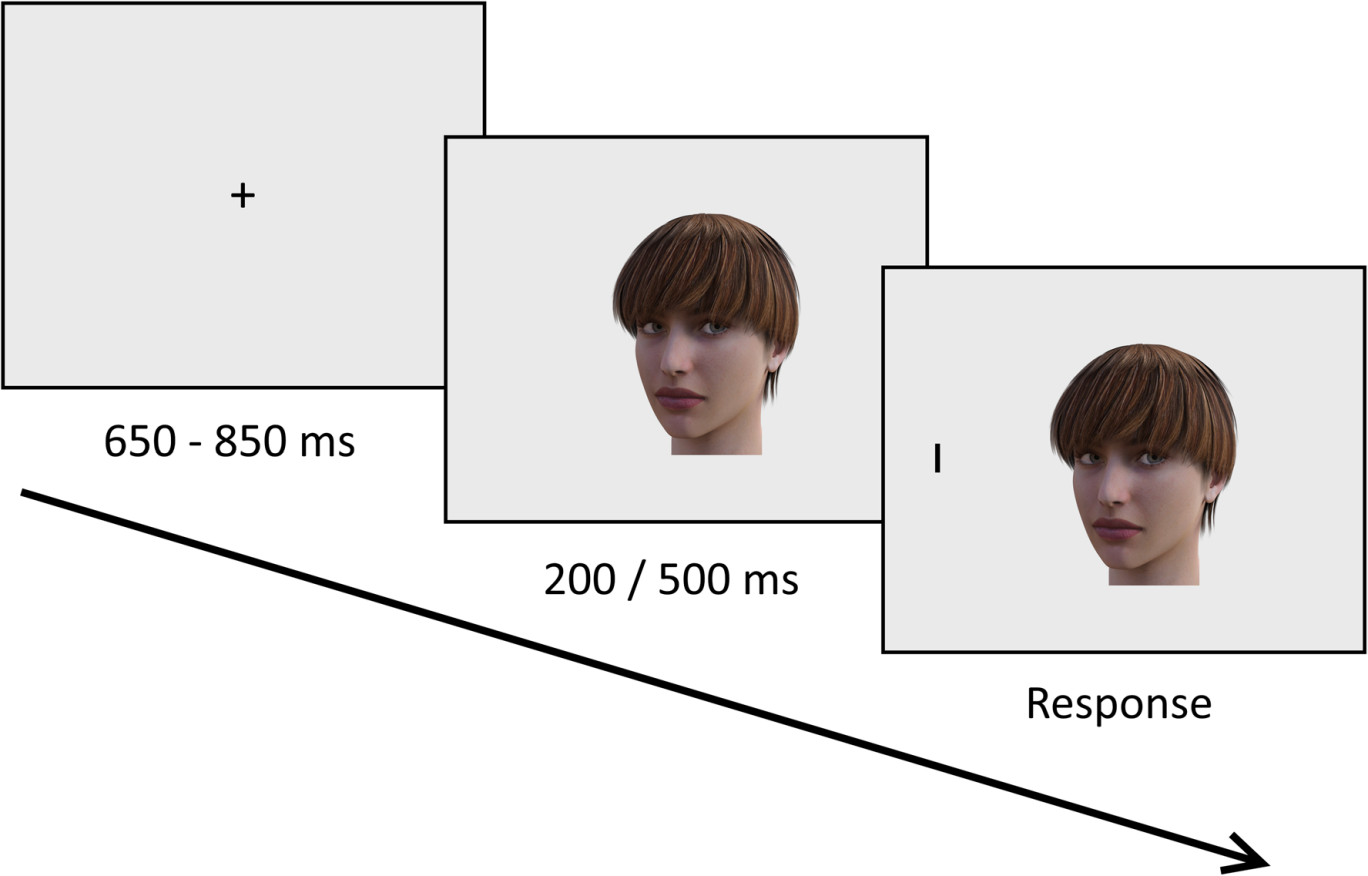
Illustration of a single trial in the attentional disengagement task. A fixation cross was displayed for 650–850 ms, after which a face stimulus (portraying direct or downward gaze) appeared on the screen. After 200 ms or 500 ms (SOA), the target stimulus (either a horizontal or a vertical line) was displayed on the left or the right side of the face. Participants were instructed to identify the target stimulus as quickly as possible using one of two keys on the keyboard. The displayed stimuli are not to scale

Each trial in the attentional disengagement task began with showing a fixation cross in the middle of the screen. After a random delay between 650 ms and 850 ms, the fixation cross was replaced with the face stimulus. The stimulus was positioned so that both eyes were at the same distance from the center of the screen. After a stimulus onset asynchrony (SOA) of 200 ms or 500 ms, the target stimulus appeared on either the left or the right side of the face (15.5° from the center of the screen). Participants were instructed to press the corresponding response key as fast as possible while trying not to make mistakes. If the participant did not respond within 2000 ms, the next trial was presented. The interstimulus interval was a random duration between 800 ms and 1200 ms. See Fig. 1 for an illustration of a single trial.

Participants completed two blocks of trials, each containing two of each possible combination of the factors of face identity, gaze direction, SOA, target stimulus, and target side. This resulted in two blocks of 128 trials each. The trial order was pseudorandomized so that there were no more than four successive repetitions on any of the factors. In one of the blocks, horizontally flipped face stimuli were displayed (order counterbalanced across participants). In between the blocks, participants were allowed to take a short break.

### Procedure

Participants arrived in the laboratory in groups of three. They were instructed by a female experimenter, blind to the condition each participant was assigned to. As a cover story, participants were told the study was about “mental visualization” and attention. They were told that they would do a mental visualization task, followed by an attention task. To enhance the cover story, participants filled in a bogus mental visualization questionnaire in the beginning of the experiment. After this, they completed a 16-trial practice block of the attentional disengagement task, followed by the Cyberball manipulation, described above. The instructions for the game were presented on the computer screen. Participants in the exclusion and inclusion conditions were told that the game would be played with the other two participants present in the laboratory via a local area network. In reality, the other characters in the game were controlled by the computer.

After the manipulation, we evaluated its effectiveness by administering a six-item questionnaire measuring fulfillment of basic social needs of control (“I felt I had the ability to significantly alter events”), meaningful existence (“I felt important”), belonging (“I felt rejected”), and self-esteem (“I felt insecure”), as well as positive mood (“I felt happy”), and negative mood (“I felt angry”). The items were chosen from a questionnaire used in previous social exclusion studies (e.g., Molet, Macquet, Lefebvre, & Williams, 2013; Wirth & Williams, 2009). The questionnaire was abbreviated, because the effects of exclusion in Cyberball have been found to diminish quickly (e.g., Lyyra et al., 2017; Wesselmann, Wirth, Mroczek, & Williams, 2012), and thus it was important to ensure the interval between the manipulation and the attentional disengagement task was as short as possible. We reverse-scored the basic need scores when necessary, and averaged them to calculate a basic need satisfaction score for each participant (*α* = 0.78). The participants also rated the amount of pain they were experiencing during the game on a 0–100 scale. As a manipulation check, they assessed what percentage of all throws in the game was made by them. After the questionnaire, participants performed the attentional disengagement task, described earlier.

After completing the task, we measured participants’ awareness of the deception in the Cyberball manipulation with a funnel-type suspicion questionnaire (the method has been previously used in Syrjämäki, Lyyra, Peltola, & Hietanen, 2017, Experiment 2). Participants typed out their answers to six questions, which started with vague questions about the experiment and ended with asking explicitly about their suspicions. We inferred that the more suspicious the participants were, the more likely they would voice their suspicions, even spontaneously to vague questions. The questions were as follows: (1) How did you feel about the experiment? (2) What do you think the experiment was about? (3) What do you think was the purpose of the ball game you played? (4) Was there anything confusing or odd about the ball game? (5) Do you think there was something about the ball game the experimenter did not tell you about? If yes, what was it? (6) If the experimenter would now tell you that she misled you with something about the ball game, what do you think she would tell you? After all participants were finished with the questionnaire, they were thoroughly debriefed, rewarded, and thanked for their participation.

### Data analysis

#### Suspicion

Each item in the suspicion questionnaire was scored 1 if the participant indicated awareness that the course of the Cyberball game was predetermined, or that the game was not played with the other participants. An item was scored 0 if the participant did not indicate such awareness. Thus, we received an ordinal scale suspicion score, ranging from 0 to 6 for each participant.

#### Data exclusions

From the total sample of 74 participants, we excluded 12 participants before the analyses. Before analyzing the data, we decided to remove all participants who received suspicion scores of 3 or higher (11 participants, 2 in the inclusion group, 9 in the exclusion group), as we considered them aware of the deception in Cyberball. Exclusion of suspicious participants did not influence the statistical significance of the analyses. Finally, we excluded one participant (in the control group) as an outlier. For this participant, the difference in response times between direct and downward gaze trials in the attentional disengagement task was very large (41 ms longer for direct gaze trials, more than three standard deviations higher than the mean difference in the sample).

#### Attentional disengagement task

For analysis of the response times, we first removed all trials in which participants did not respond (< 0.1% of all trials), trials with incorrect responses (3.8% of all trials), and trials with response times (RTs) not within 2.5 SD from the individual mean (2.6% of the remaining trials). We then calculated individual mean RTs for each combination of SOA, Gaze Direction, and Block Position. For the statistical analyses, we performed a square root transformation to correct for non-normal distribution of the data. For the sake of clarity, untransformed values are presented in the figures and text in the “Results” section. For analysis of the error rates, we calculated the total number of incorrect responses in each combination of SOA, Gaze Direction, and Block Position for each participant. For the statistical analyses, these values were square root transformed to reduce skewness. For the sake of clarity, untransformed values are presented in the “Results” section.

## Results

### Questionnaires

The means and standard deviations for manipulation check, basic need, mood and pain scores for each experimental group, as well as statistics for the between-group comparisons are presented in Table 1. Because the data violated normality assumptions of parametric tests, the comparisons were conducted using non-parametric Kruskal–Wallis tests, and the follow-up pairwise comparisons using Mann–Whitney *U* tests.

**Table 1:** Manipulation check, basic need, mood, and pain scores for each experimental group, and statistics for the between-groups comparisons

	Exclusion *M* (SD)	Inclusion *M* (SD)	Control *M* (SD)	Kruskal–Wallis test		Pairwise comparisons					
						Exclusion-inclusion		Exclusion-control		Inclusion-control	
				*χ*^2^(2)	*p*	*U*	*p*	*U*	*p*	*U*	*p*
Manip. check	11.0 (6.1)	36.6 (8.0)	83.3 (31.2)	42.84	< 0.001	0.50	< 0.001	29.00	< 0.001	45.00	< 0.001
Basic needs	2.01 (0.84)	3.85 (0.65)	3.46 (0.78)	30.92	< 0.001	29.00	< 0.001	40.50	< 0.001	136.50	0.054
Pos. mood	2.14 (1.06)	3.67 (1.24)	3.10 (1.21)	14.49	0.001	82.50	< 0.001	116.00	0.011	151.00	0.108
Neg. mood	2.29 (1.19)	1.10 (0.30)	1.20 (0.52)	21.30	< 0.001	85.50	< 0.001	93.50	0.001	197.50	0.556
Pain	24.4 (23.3)	1.5 (3.8)	6.0 (16.8)	17.11	< 0.001	82.50	< 0.001	97.50	0.002	197.50	0.685

Manipulation check and pain scores are on a 0–100 visual analogue scale; basic need and mood scores are on a 1–5 Likert scale; pairwise comparisons done with Mann–Whitney *U* test

To summarize, these results show that participants experienced the social exclusion and inclusion manipulation as intended. Participants were aware of their inclusionary status, as the manipulation checks showed that the control group reported making a larger proportion of the throws than the inclusion or the exclusion groups, and the inclusion group reported making a larger proportion of the throws than the exclusion group. Exclusion also elicited the expected affective responses: participants in the exclusion group reported less basic need satisfaction and positive mood, and more negative mood and pain than either the inclusion or the control group. There were no differences between the inclusion and control groups on these measurements, although the difference in basic need satisfaction was approaching statistical significance.

### Response times in the attentional disengagement task

**Fig. 2:**
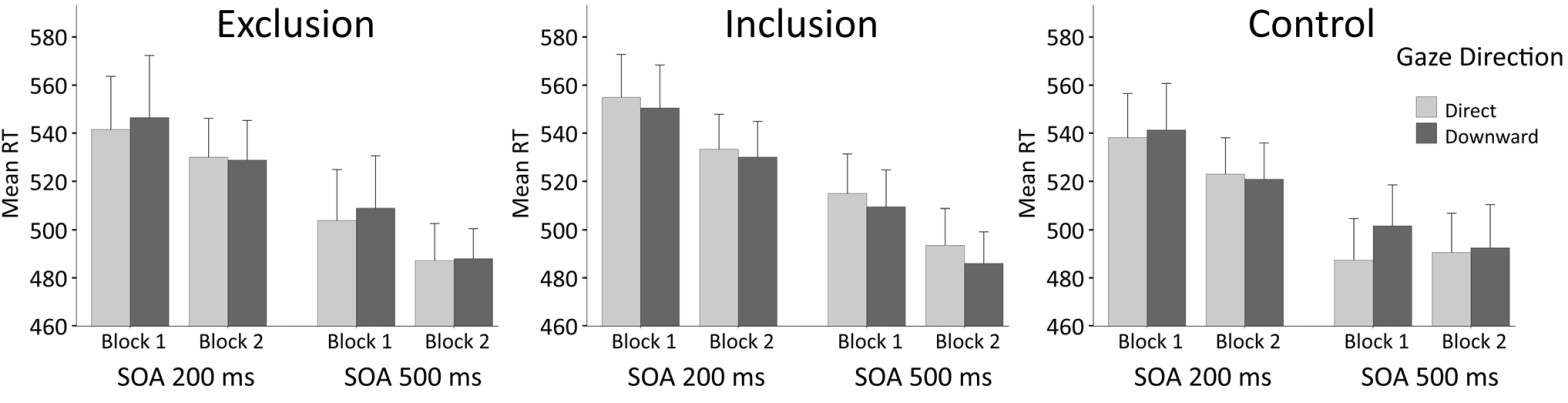
Mean response times in milliseconds in each condition. The error bars stand for standard error of the means

The response time data were analyzed using a 3 (inclusionary Status: inclusion/exclusion/control; between subjects factor) × 2 (gaze direction: direct/down; within subjects factor) × 2 (SOA: 200 ms/500 ms; within subjects factor) × 2 (block position: block 1/block 2; within subjects factor) mixed-design ANOVA, with response time (RT) as the dependent variable. For RTs divided by each of these factors, see Fig. 2.

The analysis revealed a significant effect of SOA (*F*(1, 59) = 235.42, *p* < 0.001, *η*_*p*_^*2*^ = 0.80). RTs were longer for trials at a SOA of 200 ms (*M* = 536.7, SD = 77.7), as compared to trials at a SOA of 500 ms (*M* = 497.0, SD = 71.3). Shortening of the RTs as a function of the SOA is a typical finding that reflects subjective expectancy, among other things (for a review, see Niemi & Näätänen, 1981). The main effect of Block Position was also significant (*F*(1, 59) = 10.91, *p* = 0.002, *η*_*p*_^*2*^ = 0.16). RTs were longer in block 1 (*M* = 525.0, SD = 85.3) than in block 2 (*M* = 508.7, SD = 66.2), suggesting that performance in the task improved with repetition. No main effects of gaze direction or inclusionary status were found (*p*s > 0.73).

**Fig. 3:**
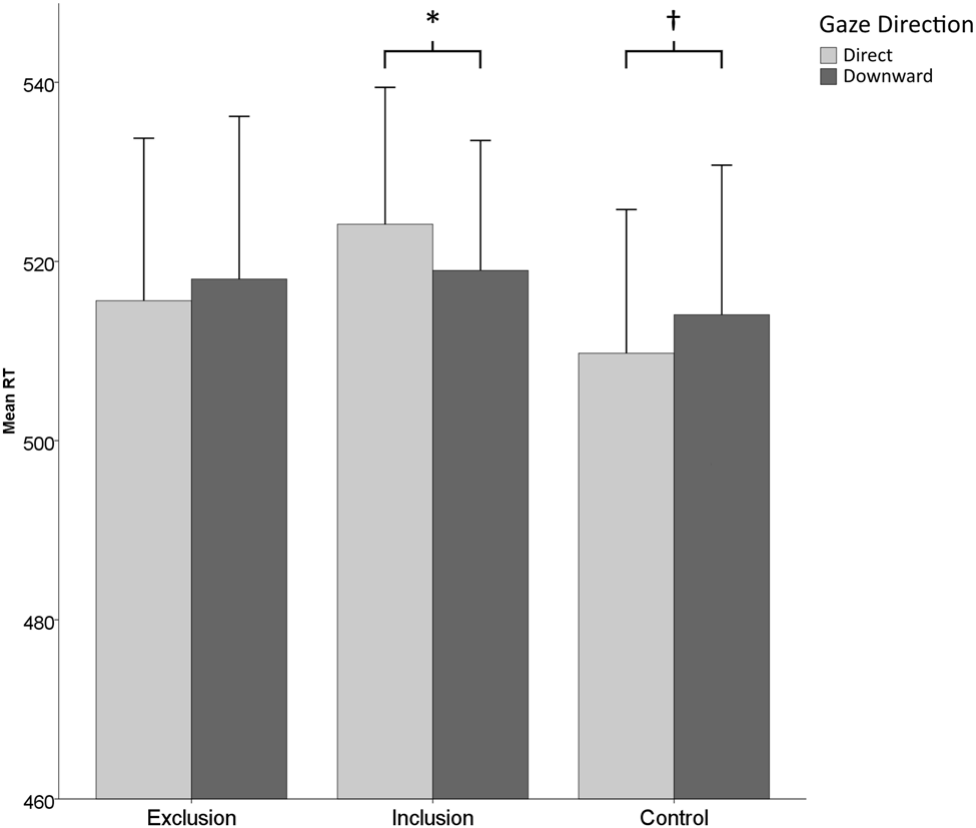
Mean response times in milliseconds in each experimental group on direct and downward gaze trials, averaged over the two SOAs and blocks. The error bars stand for standard error of the means. *p < 0.05, ^†^*p* < 0.10

The most important finding was an interaction between inclusionary status and gaze direction (*F*(2, 59) = 3.97, *p* = 0.024, *η*_*p*_^*2*^ = 0.12; see Fig. 3). To break down this interaction, we conducted a series of *t* tests. They revealed that, in the exclusion and control groups, there were no statistically significant differences between RTs in direct and downward gaze trials (exclusion group: *t*(20) = 0.77, *p* = 0.452, *d* = 0.16; control group: *t*(19) = 1.89, *p* = 0.074, *d* = 0.44), whereas the RTs were longer for direct than downward gaze trials in the inclusion group (*t*(20) = 2.41, *p* = 0.026, *d* = 0.56). No between-group differences for RTs in direct or downward gaze trials were found (*p*s > 0.50). We found no other interactions (highest *F* was for inclusionary status × SOA × block position interaction, *F*(2, 59) = 2.18, *p* = 0.123, *η*_*p*_^*2*^ = 0.07).

### Error rates

For analysis of the error rates, we conducted a similar mixed-design ANOVA as in the analysis of the RT data. The only statistically significant effect was an interaction between gaze direction and SOA (*F*(1, 59) = 5.34, *p* = 0.024, *η*_*p*_^*2*^ = 0.08; all other *p*s > 0.08). *t* tests revealed that, at the SOA of 200 ms, participants made more errors on direct gaze trials (*M* = 1.40 errors, SD = 1.18) compared to downward gaze trials (*M* = 1.15 errors, SD = 1.38; *t*(61) = 2.21, *p* = 0.031, *d* = 1.26). Participants also made more errors on direct gaze trials at the SOA of 200 ms than at the SOA of 500 ms (*M* = 1.15 errors, SD = 1.26; *t*(61) = 2.19, *p* = 0.032, *d* = 0.43). There were no differences in the number of errors between direct gaze trials at the 500-ms SOA, downward gaze trials at the 500-ms SOA (*M* = 1.22 errors, SD = 1.27), and downward gaze trials at the 200-ms SOA (*p*s > 0.33). These results suggest that participants were especially prone to make errors on direct gaze trials at the short SOA, possibly because direct gaze takes up the perceiver’s cognitive resources (see Conty, Gimmig, Belletier, George, & Huguet, 2010), consequently hindering performance in tasks that require rapid deployment of cognitive resources.

## Discussion

In the present study, we investigated whether the attentional holding effect of direct gaze (see Senju & Hasegawa, 2005) would be enhanced by social exclusion, and possibly also by social inclusion. We hypothesized that participants in all groups would be slower to identify the target stimuli when presented with a face portraying direct gaze relative to downward gaze, but more importantly, we hypothesized that this difference in response times would be larger among socially excluded participants than in the non-social control group. Neither of these hypotheses were supported. The results showed that, in the control and exclusion groups, the response times tended to be shorter for direct gaze trials than downward gaze trials. We also investigated the possibility that social inclusion would delay the disengagement of attention from faces with direct gaze. Consistent with this, we found that, in the inclusion group, the response times were significantly longer for direct gaze trials than downward gaze trials. In summary, we received no support for that direct gaze would typically delay attentional disengagement, and that this delay would be increased after an exclusion manipulation. Instead, we observed that it was the inclusion manipulation which caused delayed disengagement of attention from faces with direct gaze compared to downward gaze.

### Exclusion and attentional disengagement

As noted above, social exclusion did not influence attentional disengagement from direct gaze, in the present study. The exclusion manipulation as such was effective, as exclusion, compared to inclusion and the non-social control group, lowered mood and satisfaction of basic social needs, and increased self-reported pain, as in earlier research (e.g., Hartgerink et al., 2015; Williams et al., 2000; Wirth, Lynam, & Williams, 2010). Several researchers have suggested that exclusion increases the level of attention allocated to socially salient information (e.g., Pickett, Gardner, & Knowles, 2004; Shilling & Brown, 2016), and previous research has found increased attention toward affiliative cues, such as smiling faces, among excluded participants (e.g., DeWall et al., 2009). Just like facial expressions, also other people’s gaze is an important cue that excluded individuals use to navigate in their social environment. This is evidenced by findings that exclusion alters gaze direction judgments according to the individual’s motivational states (Lyyra et al., 2017; Syrjämäki et al., 2018), and that reflecting on exclusion, compared to inclusion, enhances attention shifts toward the direction of another person’s gaze (Wilkowski et al., 2009). Nevertheless, the exclusion manipulation did not delay the disengagement of attention from direct gaze. Perhaps excluded individuals do not maintain their attention in faces with direct gaze, because seeing direct gaze may not reduce the affective distress elicited by exclusion (see Syrjämäki et al., 2017). Attending to smiling faces, on the other hand, could be an effective way of regulating one’s affective state and therefore many people may have learned to habitually direct their attention toward smiling faces, and maintain their attention in these cues, as a response to exclusion.

Another possible interpretation of our finding is that exclusion does not exert its influence at the attentional disengagement stage. Previous research has investigated how exclusion modulates attentional shifts toward facial expressions (e.g., Tanaka & Ikegami, 2015), and fixation times to emotional faces (e.g., DeWall et al., 2009, Experiments 2–3), as well as performance in visual search for different facial expressions (e.g., DeWall et al., 2009, Experiment 1). However, the current experiment is the first to investigate how exclusion modulates attentional disengagement from a social cue^1^. Thus, it is possible that exclusion facilitates attentional shifts toward affiliative cues and engagement of attention with these cues (see DeWall et al., 2009), but does not slow down disengagement from them. Of course, we cannot draw firm conclusions because there are significant differences between this and earlier studies, such as in the types of stimuli used (emotional faces versus faces with different gaze directions). Future research should investigate whether exclusion modulates the tendency to shift attention toward faces with direct gaze (e.g., Böckler et al., 2014; von Grünau & Anston, 1995), and disengagement of attention from faces showing different facial expressions. This would provide a more detailed understanding of the time-course of the effects of exclusion on attention to different types of social cues.

It should be noted that, based on the present study, we cannot conclusively determine that exclusion did not modulate attentional disengagement from faces, in general, even though there were no differences in the response times between the groups. A limitation of the present experiment is that we did not include non-social control stimuli in the attentional disengagement task. Exclusion could have speeded up response times to targets generally, while simultaneously it may have slowed down attentional disengagement from face stimuli specifically. For instance, it has been shown that exclusion increases autonomic arousal (see Kelly, McDonald, & Rushby, 2012), which could generally speed up reaction times. If exclusion simultaneously slowed down disengagement of attention from faces, regardless of their gaze direction, these two opposite effects could have canceled each other out, so that no effect of the manipulation on response times was observed. However, based on previous research, there is no reason to assume that exclusion modulated attentional disengagement from all faces. No study to date has shown that excluded individuals allocate increased levels of attention to all types of face stimuli, but instead they tend to allocate more attention to specific types of faces, such as those who are portraying a smiling expression (e.g., DeWall et al., 2009; Tanaka & Ikegami, 2015).

### Inclusion and attentional disengagement

Interestingly, and somewhat surprisingly, our results showed that social inclusion had an effect on attentional disengagement, as the response times were longer in trials with direct gaze compared to downward gaze, when the attentional disengagement task followed an inclusive social interaction, but not after social exclusion or a non-social control task. This result suggests that social inclusion does not only increase interest in mating behavior (Brown et al., 2009; Sacco et al., 2011), but it also modulates allocation of attention to social cues. The finding was surprising, however, as we originally predicted that exclusion would be more likely than inclusion to influence attentional disengagement. One possible explanation to this finding is that social inclusion activated affiliation-related cognitive processes, which modulated responses to direct gaze. A recent study suggested that the experiential responses evoked by direct gaze are particularly powerful when participants have been primed with affective sentences related to positive social interactions or interactions involving the self, relative to negative interactions or interactions involving other people, respectively (McCrackin & Itier, 2018). Similarly, an inclusive social interaction could activate affiliation-related cognitive processes, and cause the individual to experience direct gaze as a particularly salient cue. Thus, increased allocation of attention to faces portraying direct gaze would make it difficult to disengage attention from the face.

An alternative possibility is that activating cognitive contents related to social inclusion increased attention to stimuli belonging to this category of social behavior. For instance, it has been reported that participants who wrote about a specific ethnic group showed faster visual search for faces belonging to that group compared to participants who had written about a different ethnic group (Chiao, Heck, Nakayama, & Ambady, 2006). Similarly, being included in a social interaction could cause the individual to allocate more attention to affiliative stimuli, and consequently delay disengagement of attention from these stimuli.

Future research could directly test whether a priming effect explains the delayed attentional disengagement from direct gaze. For instance, participants could be primed with sentences related to social acceptance and with non-social control sentences followed by measurements of attentional disengagement from direct and downward gaze. It would be expected that direct gaze, compared to downward gaze, would hold attention only when preceded by primes related to affiliation. Furthermore, if the effect was caused by a prime activating cognitive processes related to the category of the perceived stimulus, then a similar effect on attentional disengagement might occur by priming different types of categories of stimuli as well. Primes related to, for instance, animals, might lead to delayed attentional disengagement from pictures of animals, compared to control stimuli such as pictures of plants. This hypothesis could be investigated in future research to determine if the effect of social inclusion on attentional disengagement from direct gaze reflects a typical response to various types of primes and stimuli, or if this response is specific to affiliative social cues followed by affiliation-related affective priming.

Even though we have interpreted our results in the context of attentional engagement, is it possible that the observed difference in response times between direct and downward gaze trials can be explained by some other effects? This seems improbable, as several alternative explanations can be ruled out. First, we can rule out the possibility that the difference in response times would reflect the effect of gaze direction on autonomic arousal. It has been demonstrated that gaze direction in pictures of faces does not influence autonomic arousal, unlike gaze direction in live faces (e.g., Hietanen et al., 2008; Pönkänen, Peltola, & Hietanen, 2011). Even if the face stimuli had influenced autonomic arousal in this experiment, direct gaze would have been expected to increase arousal, which in turn, should have led to shortening rather than lengthening of the response times. Second, the effect was not likely caused by the manipulation altering socially included participants’ perceptions of gaze directions. Previous research has shown that social exclusion modulates judgments of others’ gaze directions, but importantly, social inclusion does not (Lyyra et al., 2017; Syrjämäki et al., 2018). Thus, if the modulation of response times would have reflected changes in perception of gaze directions, we should have observed the effect of gaze in the social exclusion group, rather than in the social inclusion group. Finally, it is unlikely that the difference in response times was due to included participants having difficulties in making a saccade from the faces to the target stimuli, rather than having difficulties in disengaging attention as such. Attentional shifts precede saccades (Zhao, Gersch, Schnitzer, Dosher, & Kowler, 2012), and thus if the direct gaze stimuli only delayed oculomotor disengagement, but not attentional disengagement, participants’ attention would have shifted to the target stimuli equally quickly on both types of trials. It is clear from previous research that responding to peripheral stimuli is possible without moving eyes away from a central stimulus (e.g., Hermens, 2015), and in the current study, the visual difference between the two target stimuli was discernible while fixating on the centrally presented face. Because participants were attempting to respond as quickly as possible, it is unlikely that they would have deliberately waited for the saccade before responding if their attention was already focused on the target stimulus.

### Non-social control condition

From a methodological standpoint, the most important implication of the current study is that social inclusion is not always a suitable control condition when investigating the effects of social exclusion using the Cyberball manipulation. In a typical experiment using this manipulation, any differences between the exclusion and the inclusion groups are inferred to reflect effects of exclusion (Hartgerink et al., 2015). A growing body of evidence suggests that the effect of the manipulation on affect is indeed driven by exclusion and not inclusion (Dvir et al., 2018; Riva et al., 2014; Syrjämäki et al., 2018). The current results are consistent with these findings, as we found no statistically significant differences between included participants and the control group in basic need satisfaction, mood, or self-reported pain (although in basic need satisfaction, the difference was approaching statistical significance). Furthermore, previous research also shows that the effects of the manipulation on gaze direction judgments (Syrjämäki et al., 2018), and compliance (Riva et al., 2014) are caused by exclusion, and not inclusion. However, the current study shows that inclusion, but not exclusion in Cyberball modulated disengagement of attention from faces with direct gaze, compared to downward gaze. This shows that some of this manipulation’s effects are driven by inclusion and, therefore, future research should use non-social control groups to firmly show that differences between exclusion and inclusion groups are driven by exclusion (for a similar argument, see Brown et al., 2009). However, we do not imply that previously reported effects of exclusion manipulations on attention are driven by inclusion, as many studies on this issue have used control groups other than social inclusion (e.g., Buckner et al., 2010; DeWall et al., 2009).

We propose that the non-social task used in this experiment provides an appropriate and convenient control condition for future studies investigating the effects of social exclusion and social inclusion using Cyberball. Other types of control manipulations have been used as well, but the manipulation used in the current study has a few strengths over them. Riva et al. (2014) instructed participants to mentally visualize natural scenery, and Dvir et al. (2018) showed participants pictures of trees, which they were instructed to mentally visualize and to click on them with a mouse to emulate the motor actions done during Cyberball. Like these tasks, the currently used control task was devoid of any social interaction, but unlike in these other control tasks, participants performed identical actions as in the standard version of Cyberball, i.e., mouse clicks to throw a ball in a simple computer game. Moreover, in this task, participants were not led to mentally visualize nature, which might evoke unwanted responses. Even passive viewing of natural scenes can improve the perceiver’s affective state (e.g., Ulrich et al., 1991) and influence recognition of affectively congruent facial expressions (Hietanen, Klemettilä, Kettunen, & Korpela, 2007).

### Direct gaze and attentional disengagement

An important finding of this study was that, in general, there were no significant differences in response times in identification of the target stimuli between direct and downward gaze trials, suggesting that direct gaze did not hold observers’ visuospatial attention. A few other recent studies have also found convergent evidence. Dalmaso et al. (2017) reported that only in one out of three experiments, delays in saccades from faces to peripheral stimuli were longer in the context of direct gaze compared to downward gaze. In the other two experiments, the delays were similar for the two conditions (but see Ueda et al., 2014, for a finding that saccadic latencies were longer from faces suddenly shifting eyes toward the perceiver, compared to faces shifting gaze upward or downward). Strikingly, another recent study found that eye contact with a live confederate enhanced, rather than impaired, attentional disengagement as measured by manual response times to peripheral stimuli (Hietanen et al., 2016). In other words, the result was opposite to what Senju and Hasegawa (2005) reported. The authors suggested that the engagement of visual attention by direct gaze was possibly overridden due to increased physiological arousal elicited by eye contact with a live confederate and that the increased arousal also facilitated perceptual-motor processes involved in discriminating and responding to the visual targets.

A comparison between the original experiment by Senju and Hasegawa (2005) and the studies mentioned above is somewhat problematic because the stimuli (Hietanen et al., 2016) or the behavioral measurements (Dalmaso et al., 2017) differed from those used in the Senju and Hasegawa study. The current study provides the first reported replication attempt of the original finding, measuring manual response times and using pictorial face stimuli as in the original study. Critically, we included a 500-ms SOA condition, in which the difference in response times between the two gaze conditions was found earlier. Notably, there were differences between the tasks in the two studies as well. For instance, the current study used computer-generated face stimuli, whereas the original study used photographs. However, it seems unlikely that this difference explains the conflicting results, as previous studies have reported effects of direct gaze on saccadic latencies even when using unrealistic schematic faces (Ueda et al., 2014). We cannot, of course, rule out the possibility that the discrepant results are explained by some other difference between the tasks (such as the 3°-difference in the positioning of the target stimuli, or difference in the task demands, i.e., identification versus detection of the target stimuli), but we have no reason to believe this is the case.

One potential, albeit unlikely, explanation for why there was no general effect of direct gaze on attentional disengagement is that participants did not perceive the direct gaze stimuli as actually portraying direct gaze. Due to the Wollaston effect, an objectively direct gaze can appear averted when the head is rotated (see Langton, Watt, & Bruce, 2000). However, we believe it is unlikely that the Wollaston effect distorted perception of gaze directions in this experiment. While we did not directly assess whether participants perceived the faces as portraying direct gaze as intended, the answers to the open-ended questions in the post-experiment suspicion questionnaire suggest that they did. Nine participants explicitly indicated that the faces were occasionally portraying direct gaze, for instance by referring to the faces as “staring faces”. Eleven more participants referred to the gaze directions more vaguely, so that it was not possible to determine where they thought the faces were looking at. Importantly, however, no participant explicitly indicated that the faces were portraying averted gaze, suggesting that the Wollaston effect did not influence gaze direction perception in this experiment.

It is also extremely unlikely that the failure to replicate the earlier result was due to low statistical power. Our experiment had a significantly larger sample size than the original study (62 participants after all data exclusions in the present study, 7 participants in the original study). Thus, our results strongly suggest that direct gaze does not generally slow down attentional disengagement from the face. However, while the experiment was well-powered to detect a general effect of direct gaze on attentional disengagement, it had less statistical power to detect effects of the exclusion and inclusion manipulations on the attentional disengagement, and thus findings regarding the effects of these manipulations should be interpreted more cautiously.

## Conclusion

In the present study, we found no evidence that direct gaze would generally hold a perceiver’s visuospatial attention, or that social exclusion would slow down attentional disengagement from direct gaze. Surprisingly, we found that a social inclusion manipulation modulated attentional disengagement; following inclusion, disengagement of attention was slower from faces with direct gaze compared to downward gaze.

## Footnotes

1 DeWall et al. (2009, Experiment 4) investigated the effects of exclusion on attention to different facial expressions using a dot-probe task. They interpreted their result as indicating that exclusion modulated attentional disengagement from a smiling face, but not the speed at which attention was engaged with a smiling face. However, the dot-probe task used in that experiment only reveals which of two stimuli (a neutral face or an emotional face) attention was focused on at the time the target probe was presented (1 s after presentation of the faces in that experiment), and thus it cannot distinguish between effects at different stages of attentional deployment (see Cooper & Langton, 2006). Thus, the current experiment is in fact the first to specifically investigate the effects of exclusion on attentional disengagement from a social stimulus.
